# Exploring subthreshold processing for next-generation TinyAI

**DOI:** 10.3389/fncom.2025.1638782

**Published:** 2025-07-31

**Authors:** Farid Nakhle, Antoine H. Harfouche, Hani Karam, Vasileios Tserolas

**Affiliations:** ^1^Department of Computer Science, Temple University, Japan Campus, Tokyo, Japan; ^2^Unité de Formation et de Recherche en Sciences Économiques, Gestion, Mathématiques, et Informatique, Université Paris Nanterre, Nanterre, France; ^3^Faculty of Business and Economics, American University of Science and Technology, Beirut, Lebanon

**Keywords:** dendritic processing, energy efficiency, graded activations, hybrid analog-digital systems, neuromorphic computing, subthreshold processing, sustainable AI design, TinyAI

## Abstract

The energy demands of modern AI systems have reached unprecedented levels, driven by the rapid scaling of deep learning models, including large language models, and the inefficiencies of current computational architectures. In contrast, biological neural systems operate with remarkable energy efficiency, achieving complex computations while consuming orders of magnitude less power. A key mechanism enabling this efficiency is subthreshold processing, where neurons perform computations through graded, continuous signals below the spiking threshold, reducing energy costs. Despite its significance in biological systems, subthreshold processing remains largely overlooked in AI design. This perspective explores how principles of subthreshold dynamics can inspire the design of novel AI architectures and computational methods as a step toward advancing TinyAI. We propose pathways such as algorithmic analogs of subthreshold integration, including graded activation functions, dendritic-inspired hierarchical processing, and hybrid analog-digital systems to emulate the energy-efficient operations of biological neurons. We further explore neuromorphic and compute-in-memory hardware platforms that could support these operations, and propose a design stack aligned with the efficiency and adaptability of the brain. By integrating subthreshold dynamics into AI architecture, this work provides a roadmap toward sustainable, responsive, and accessible intelligence for resource-constrained environments.

## 1 Introduction

The accelerating development of artificial intelligence (AI) systems, particularly large-scale models such as large language models (LLMs), has led to remarkable advancements in machine learning and automation. However, these advancements have come at a significant cost: the energy demands of AI systems are escalating at an unsustainable rate. For instance, the training of state-of-the-art models like GPT-3 required an estimated 1,287 megawatt-hours of electricity, equivalent to the annual energy consumption of over 100 average households (Probst, 2023; Wang Q. et al., 2024). Such high energy usage highlights the inefficiencies inherent in current AI computational architectures and raises critical environmental concerns. The environmental impact of AI goes beyond energy consumption. Large-scale AI models often require extensive water resources for cooling and contribute to significant carbon emissions (Crawford, 2024; Wang Q. et al., 2024). As global efforts intensify to address climate change, AI’s burgeoning energy footprint risks undermining sustainability goals. The convergence of these challenges necessitates a paradigm shift in AI design, one that prioritizes energy efficiency without compromising computational performance.

In contrast to artificial systems, biological neural networks operate with unparalleled energy efficiency. For example, the human brain consumes approximately 20 watts of power (Racker and Kabat, 1942; Zador et al., 2023), yet it outperforms many AI systems in tasks requiring generalization and adaptability (Cohen et al., 2022). One critical mechanism underpinning this efficiency is subthreshold processing. This mode of neural computation involves continuous, graded signals below the spiking threshold, allowing neurons to perform complex calculations with minimal energy expenditure (Loyez et al., 2021; Cohen et al., 2022; Padamsey et al., 2022). Subthreshold processing enables biological systems to function effectively in energy-constrained environments, making it a compelling model for designing energy-efficient artificial systems. Despite its prominence in biology, subthreshold processing remains underexplored in AI architectures. This oversight represents a missed opportunity to leverage a proven, low-energy computational strategy for advancing AI (Loyez et al., 2021; Editorial, 2023).

In this work, we use the term “subthreshold regime” in a generalized and cross-disciplinary sense. Biologically, it refers to graded changes in membrane potential below the spiking threshold, enabling energy-efficient integration of information. In hardware, particularly in analog or neuromorphic designs, subthreshold operation often refers to transistor-level functioning at voltages below threshold, where current varies exponentially with gate voltage. In digital and algorithmic contexts, where physical voltage thresholds do not directly apply, operating in a subthreshold regime denotes adopting design principles that emulate this graded, low-power computation: smooth activation functions, limited dynamic ranges, sparse and localized processing, and event-driven logic. These strategies mirror the analog integration and selective activation seen in biological neurons. Thus, across system levels, “subthreshold” becomes a unifying principle guiding the shift toward energy-proportional, context-sensitive AI. The potential of subthreshold processing to revolutionize AI extends beyond energy savings; by incorporating graded activation functions, inspired by the smooth, continuous nature of biological signals, AI systems could achieve more nuanced computations (Cohen et al., 2022). While some AI systems use smooth activations like sigmoid or softplus in specific contexts, subthreshold-inspired designs offer a new avenue for more biologically realistic and energy-efficient mechanisms. From an algorithmic standpoint, subthreshold processing can be abstracted as smooth, continuous transformations of input signals, captured through activation functions that avoid hard thresholds and instead reflect graded, contextual responsiveness. This conceptual bridge enables AI models to emulate the analog computation of neurons, even when implemented in digital substrates. Such designs could reduce the reliance on binary or threshold-based activations like rectified linear unit (ReLU), which dominate current AI architectures, while simultaneously decreasing energy costs (Loyez et al., 2021; Editorial, 2023). Additionally, the hierarchical processing observed in dendrites, a hallmark of neuronal computation, offers a blueprint for creating sparse and localized networks that minimize redundancy (Loyez et al., 2021; Cohen et al., 2022). This could further enhance the computational efficiency and scalability of AI systems, particularly in edge computing applications (Loyez et al., 2021; Wang Q. et al., 2024). Hybrid analog-digital systems also stand out as a promising direction. These systems, which blend the continuous nature of analog computation with the precision of digital processing, could emulate subthreshold dynamics more effectively than traditional digital architectures (Loyez et al., 2021; Editorial, 2023). Recent advances in neuromorphic hardware and AI design, such as spiking neural networks (SNNs) operating in subthreshold regimes, underscore the feasibility of implementing these principles in real-world systems (Loyez et al., 2021). The exploration of subthreshold-inspired computation aligns seamlessly with the goals of TinyAI, a paradigm that emphasizes compact, efficient, and sustainable AI systems. TinyAI is particularly critical for the proliferation of AI in energy-constrained environments such as mobile devices, IoT sensors, and remote edge systems (Wang Q. et al., 2024). Unlike their large-scale counterparts, which rely on centralized data centers and extensive computational resources, TinyAI systems aim to decentralize and distribute intelligence in a resource-efficient manner. This transition could mitigate the environmental costs associated with traditional AI while expanding its accessibility and applicability (Editorial, 2023; Crawford, 2024). Furthermore, the integration of subthreshold principles into AI design could redefine the hardware-software co-design landscape. Neuromorphic chips, which mimic the architecture and functionality of biological neural networks, are poised to play a pivotal role in this transformation (Schuman et al., 2022; Nakhle, 2024). These chips, operating at ultra-low power, are designed to leverage the energy-efficient properties of subthreshold dynamics (Loyez et al., 2021). Advances in materials science, such as memristive devices and van der Waals materials, offer additional avenues for creating hardware optimized for subthreshold computation (Editorial, 2023). These innovations not only enhance computational efficiency but also open doors for novel applications, including real-time processing in embedded systems and adaptive learning algorithms for dynamic environments (Nakhle, 2024; Nirmal et al., 2024; Wang S. et al., 2024).

This perspective delves into the untapped potential of subthreshold processing as a transformative framework for advancing TinyAI. We go beyond isolated techniques and propose a cohesive, hierarchical design framework that aligns biological principles with computational abstractions and hardware implementations. This framework integrates pathways such as graded activation functions, dendritic-inspired hierarchical processing, and hybrid analog-digital computation to emulate the energy-efficient operations of biological neurons. Additionally, we highlight the critical role of neuromorphic and compute-in-memory (CIM) hardware in realizing these strategies, emphasizing their potential to reduce energy consumption and enable scalable, sustainable AI. Drawing from neuroscience, computer science, and hardware engineering, this cross-disciplinary synthesis presents a clear roadmap for developing biologically grounded TinyAI systems that are both energy-efficient and adaptable to real-world constraints. While our focus is on TinyAI systems, many of the energy-saving strategies explored here could also help address the unsustainable energy scaling of large models, offering benefits at both ends of the AI deployment spectrum. Ultimately, we envision a future where biologically inspired principles guide the design of AI architectures, enabling the creation of compact, scalable, and environmentally responsible technologies to meet the demands of the 21*^st^* century.

## 2 The hidden secret of subthreshold processing

Subthreshold processing, a mechanism ubiquitously observed in biological neural systems operates through continuous, graded changes in membrane potential below the spiking threshold unlike spiking activity, which involves discrete, energetically expensive action potentials. These subtle fluctuations are critical for the overall computational efficiency of biological systems, enabling neurons to integrate vast amounts of synaptic inputs with minimal metabolic cost (Zendrikov et al., 2023; Amsalem et al., 2024). The membrane potential of a neuron, even when not generating action potentials, is influenced by the summation of excitatory and inhibitory synaptic inputs. This subthreshold activity allows neurons to perform complex computations, such as integrating spatially and temporally distributed inputs, without expending the energy required for spiking. Dendrites, the branching structures of neurons, are particularly important in this regard. They process incoming signals locally through subthreshold dynamics, enabling tasks like coincidence detection, signal amplification, and non-linear integration. These local computations are crucial for higher-order brain functions, including decision-making and sensory processing (Thakar et al., 2023; Amsalem et al., 2024). Subthreshold processing also underpins the efficiency of cortical networks, which often operate in a fluctuation-driven regime. In this state, neurons remain below the threshold for spiking most of the time, relying on the balance of excitatory and inhibitory inputs to modulate their activity. This balance not only conserves energy but also enhances the network’s ability to encode and transmit information with high fidelity. Such mechanisms are particularly evident in areas like the anterior lateral motor cortex, where subthreshold dynamics contribute to preparatory activity and decision-making processes (Amsalem et al., 2024). Subthreshold activity also facilitates a seamless integration of sensory inputs, allowing organisms to respond effectively to subtle environmental changes (Sumner et al., 2006).

In computers, traditional architectures like Von Neumann-based systems rely heavily on discrete, digital computations, which are both energy-intensive and constrained by bottlenecks in data transfer between memory and processors (Zendrikov et al., 2023). Subthreshold-inspired designs, by contrast, offer a paradigm shift toward hybrid analog-digital systems capable of processing information more efficiently. Thus, translating the principles of subthreshold processing into AI presents a transformative opportunity to address the escalating energy demands of modern AI systems.

## 3 Neuron diversity and the scope of subthreshold processing

While subthreshold integration offers a biologically grounded pathway toward energy-efficient computation, it represents only one mode within a broader spectrum of neuronal processing strategies. The brain is composed of a diverse array of neuron types, each with unique morphologies, ion channel compositions, and firing behaviors that support specialized computational roles. These include fast-spiking interneurons, bursting pyramidal neurons, resonant thalamic cells, and neuromodulatory systems such as dopaminergic and cholinergic neurons, each contributing distinct energy-computation trade-offs (Llinás, 1988; Marder and Goaillard, 2006; Sterling and Laughlin, 2015). For instance, fast-spiking interneurons, such as parvalbumin-positive cells, exhibit narrow action potentials and high-frequency discharge, essential for network synchrony and gamma oscillations, but rely heavily on rapid above-threshold dynamics. In contrast, bursting neurons, common in the thalamus and hippocampus, alternate between quiescent and high-frequency states based on intrinsic membrane properties and input frequency (Izhikevich, 2000). These neurons are thought to play a pivotal role in salience detection, signal amplification, and sleep-wake transitions; functions that extend beyond pure energy minimization. Resonant neurons, such as thalamocortical relay cells, preferentially respond to inputs at specific frequencies due to membrane resonance, a mechanism critical for temporal filtering and phase-locked responses (Hutcheon and Yarom, 2000). Similarly, neuromodulatory systems influence circuit function by altering cellular excitability and synaptic plasticity over longer timescales, often through volume transmission and subthreshold modulations (Marder and Goaillard, 2006).

Despite this rich heterogeneity, the present focus on subthreshold processing stems from its central role in energy efficiency. Subthreshold dynamics avoid the metabolic costs of action potentials and enable continuous, analog-like computation, making them highly compatible with TinyAI’s low-power objectives. Furthermore, current neuromorphic and CIM platforms are increasingly capable of emulating subthreshold regimes, while faithfully reproducing fast-spiking or resonant dynamics remains more challenging due to thermal noise, timing precision, and material constraints (Du et al., 2020; Yao et al., 2020). Subthreshold-inspired activation functions also offer a tractable and differentiable pathway to integrate biological insights into gradient-based learning, a major advantage for hybrid analog-digital systems. By acknowledging the broader landscape of neuronal diversity, we underscore that subthreshold processing is not an exclusive or exhaustive paradigm but rather a strategic design anchor. Yet, it serves as an entry point for translating biological principles into practical, scalable AI systems. Future work may incorporate dynamics from resonant, bursting, or neuromodulated neurons to further enrich the architectural design of TinyAI.

## 4 Energy challenges in modern AI

The rapid scaling of AI technologies, particularly the race of LLMs that we are currently witnessing, presents profound energy sustainability challenges. These models, while revolutionary, demand substantial computational resources for their training and operation. For instance, training GPT-4 consumed over 50 GWh of electricity, equivalent to about 0.02% of California’s annual electricity usage, and marked a 50-fold increase from its predecessor GPT-3, which itself required approximately 1,287 MWh (De Vries, 2023; Li et al., 2024) Such energy demands underscore the unsustainable trajectory of current AI growth, especially as the computational requirements of AI models continue to double approximately every 10 months (Li et al., 2024).

Beyond electricity consumption, the environmental footprint of AI models includes substantial water usage, often overlooked in sustainability discussions. Training GPT-3 in Microsoft’s advanced data centers, for instance, resulted in the evaporation of approximately 700,000 liters of freshwater (Li P. et al., 2023). Moreover, global AI operations are projected to account for a staggering 4.2–6.6 billion cubic meters of water withdrawal by 2027, a figure surpassing the annual water withdrawal of several European countries (Li P. et al., 2023). This water-intensive nature of AI is exacerbated by the cooling demands of data centers, which not only utilize significant water volumes but frequently compete with local communities for scarce freshwater resources (Mytton, 2021).

A critical concern arises from the disruptive impact of AI-induced transient power demands on energy grids. Large-scale LLM training workloads can spike from cold starts to peak loads of tens of megawatts almost instantaneously, a scale previously unseen and unmanageable by traditional grid designs (Li et al., 2024). Such rapid fluctuations pose substantial threats to power grid stability, highlighting an urgent need for improved grid infrastructure and dynamic energy management solutions to accommodate these sudden and significant energy demands. Economic factors also interplay significantly with AI’s energy consumption. While AI technologies promise productivity gains, their high energy costs could ultimately constrain accessibility, limiting such powerful tools to resource-rich entities and exacerbating existing inequities in technological advancement (Schwartz et al., 2020; Strubell et al., 2020). The environmental and economic impacts of AI underscore a critical juncture: without substantial improvements in computational efficiency, hardware sustainability, and energy sourcing, the benefits of AI may become increasingly skewed toward those with substantial financial resources, undermining broader social and environmental sustainability goals (Bourzac, 2024).

Despite these daunting challenges, the situation presents significant opportunities for innovation. The development of energy-efficient hardware architectures such as neuromorphic computing, hybrid analog-digital systems, and specialized AI accelerators offers promising pathways to mitigate AI’s growing energy footprint (Bourzac, 2024; Nakhle, 2024). In addition, algorithmic advancements in model compression, pruning, quantization, and knowledge distillation techniques are becoming increasingly critical to reducing the resource intensity of AI models (Strubell et al., 2020; Nakhle, 2024). On top of those, this perspective highlights the promising role of biologically inspired approaches, such as subthreshold processing, as potential pathways to significantly further enhance energy efficiency in AI systems and address sustainability concerns.

## 5 Subthreshold-inspired pathways to advance TinyAI

Despite their common goal, i.e., processing information, biological neurons and silicon chips that run artificial neural networks (ANNs) operate with profoundly different energy strategies. Biological neurons, particularly cortical types, accumulate thousands of graded inputs in a metabolically efficient manner, emitting a spike only when the integrated voltage crosses a threshold. This enables predominantly analog, low-power computation within dendrites. In contrast, most modern digital processors, including the edge accelerators used to host and run TinyAI models, perform arithmetic using full-swing digital signals, operating well above the point where their transistors begin to conduct. These above-threshold transitions are simple to engineer but inherently energy-inefficient.

**FIGURE 1:**
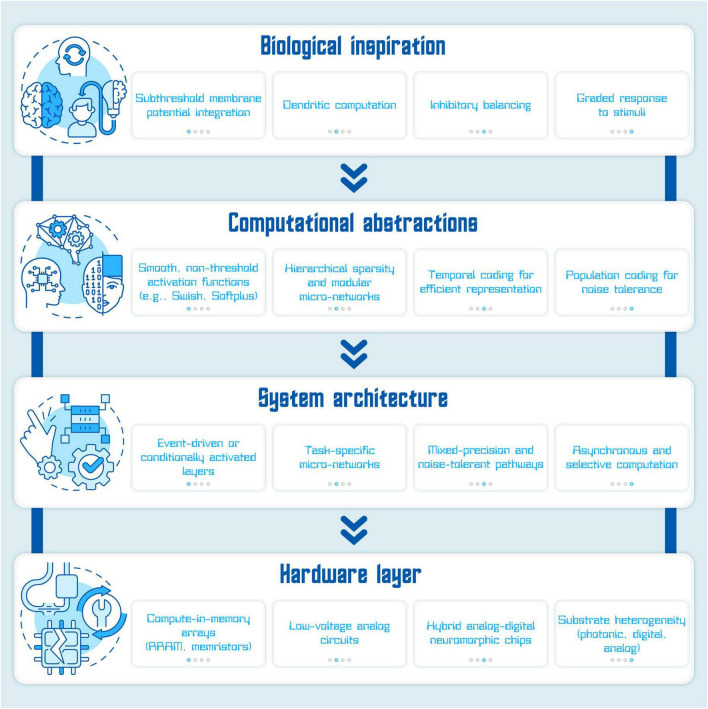
Subthreshold-inspired design stack for energy-efficient AI design. This multi-layered framework illustrates how biologically inspired mechanisms can be systematically translated into increasingly concrete computational, architectural, and hardware components for sustainable TinyAI. The top layer, Biological inspiration, draws from principles of neuronal computation observed in the brain, including subthreshold membrane potential integration, dendritic computation, inhibitory balancing, and graded responses to stimuli. These mechanisms enable high energy efficiency and context-sensitive information processing in biological systems. The second layer, Computational abstractions, translates these biological features into machine learning techniques such as smooth, non-threshold activation functions (e.g., Swish, Softplus), hierarchical sparsity, temporal coding for efficient representation, and population coding for noise tolerance. These abstractions allow artificial systems to maintain low energy usage and noise robustness while preserving expressive power. The third layer, System architecture, incorporates these abstractions into the design of AI models. Key features include event-driven or conditionally activated layers that reduce idle computation; task-specific micro-networks that localize processing; mixed-precision and noise-tolerant pathways that enable resilience in resource-constrained conditions; and asynchronous, selective computation strategies that mimic the dynamic routing capabilities of biological circuits. These elements enable TinyAI models to operate responsively with minimal energy overhead, especially in embedded or edge settings. The bottom layer, Hardware layer, consists of physical implementations that support subthreshold-compatible computation. This includes compute-in-memory arrays (e.g., memristors) that integrate memory and processing to eliminate energy-intensive data movement; low-voltage analog circuits that exploit subthreshold transistor operation for energy savings; hybrid analog-digital neuromorphic chips that combine biological realism with programmable control; and substrate heterogeneity, allowing components to be matched to their ideal physical medium (e.g., analog for static filters, digital for classifiers, photonic for high-throughput inference). Together, these hardware strategies form the physical foundation for building responsive, low-power TinyAI systems suited for real-world, energy-constrained applications.

**FIGURE 2:**
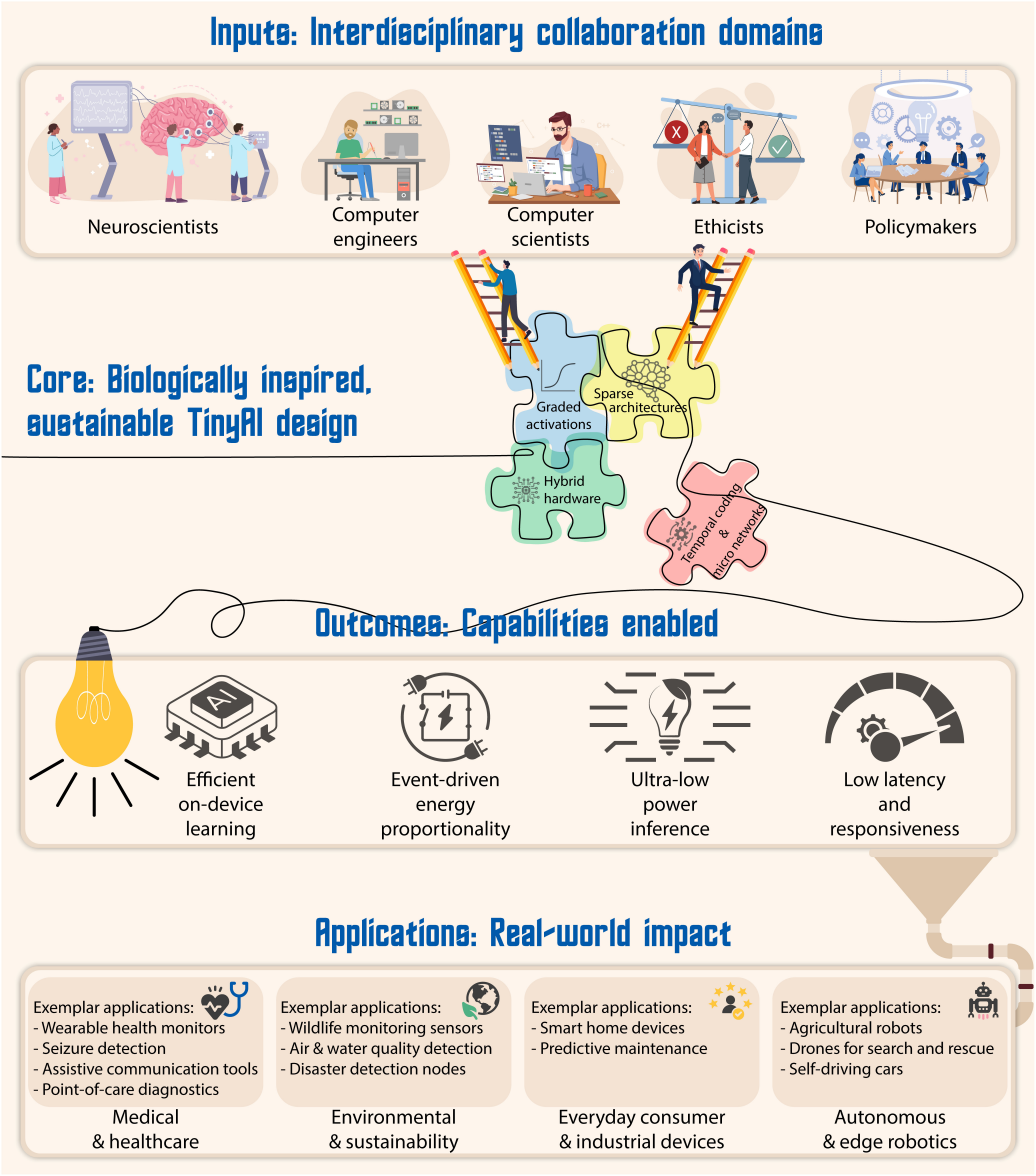
From collaboration to impact: a layered framework for sustainable TinyAI. The top layer represents interdisciplinary collaboration, with each domain contributing essential foundations such as subthreshold dynamics, hybrid hardware design, energy efficiency, and equitable access. These inputs converge into the central system layer: sustainable TinyAI, characterized by biologically inspired mechanisms like graded activation functions, hierarchical sparsity, and hybrid analog-digital hardware. This design stack enables ultra-low power, explainable, and locally adaptive intelligence. From this foundation emerge core system capabilities, such as on-device inference, energy proportionality, responsiveness, and explain ability which in turn power a diverse range of real-world applications. These include wearable medical devices, point-of-care diagnostics, environmental monitoring sensors, edge robotics, and assistive technologies.

The field of TinyAI has already achieved impressive reductions in model size and compute cost through pruning, quantization, and knowledge distillation (Nakhle, 2024). Yet, these algorithmic savings often sit atop digital hardware that remains energy-hungry at the physical layer. Much of the latent efficiency observed in biological computation, particularly the ability to process information in the subthreshold regime, is still absent in the design of AI models and hardware. To close this gap and move closer to the energy proportionality observed in biology, we propose a subthreshold-aware approach that treats low-voltage operation not as a hardware constraint to be tolerated, but as a design dimension to be deliberately explored. The perspective developed here suggests that by integrating principles of subthreshold computation into the core of AI design, at the levels of activation dynamics, computational structure, hardware substrate, compilation tooling, and time-dependent information encoding, we can open a complementary region of the efficiency landscape that current methods only brush against (Figure 1). This framework is particularly well-suited for TinyAI: systems that must operate at the edge, under tight energy and resource constraints, but still deliver capable and intelligent inference. What follows are five design strategies that connect biological mechanisms to computational techniques and implementation pathways, aiming to guide future model design toward radical, biologically inspired efficiency. To anchor this framework visually, we introduce Figures 1, 2, which synthesizes these pathways into a hierarchical design stack inspired by subthreshold computation.

### 5.1 Smooth activation dynamics for low-voltage computation

As discussed previously, most biological computations occur below the spiking threshold, in the form of small voltage fluctuations across the neuronal membrane. These continuous, analog responses are not only essential for temporal integration and contextual modulation, but also highly energy-efficient, avoiding the metabolic cost of firing an action potential. In contrast, ANNs typically rely on activation functions like the ReLU, which abruptly zeroes out negative inputs and transmits positive inputs linearly. While ReLU supports fast model training and stable gradient flow, it introduces sharp discontinuities that lead to wide internal voltage swings, making it poorly matched to low-power hardware. Replacing ReLU with smooth, continuous functions such as Swish, defined as Swish = x Sigmoid(βx), where β represents a constant or trainable parameter, provides activation dynamics that more closely mirror subthreshold biological integration. Algorithmically, these functions model how neurons accumulate input in a continuous fashion, encoding stimulus intensity over time rather than responding in a binary all-or-nothing manner. This allows neural networks to express graded sensitivity to features, reflecting the analog computation of biological neurons in software. Notably, Swish not only improves optimization in deep networks but also enhances final accuracy, raising top-1 ImageNet performance by approximately 0.9 percentage points in otherwise identical architectures (Ramachandran et al., 2017). Crucially, its narrow dynamic range enables inference engines to operate at lower voltages, reducing energy per operation without loss of representational fidelity. However, implementing these smooth functions on analog or CIM hardware introduces challenges such as device mismatch, thermal noise, and limited signal resolution, which can distort narrow dynamic ranges. These issues must be managed through noise-tolerant training and activation-range calibration.

This compatibility becomes even more critical when deploying models to analog or mixed-signal CIM architectures, where the energy cost of signal transitions and data movement dominates total power consumption. By physically integrating computation into the memory array, CIM eliminates the need to shuttle activations and weights between memory and processor, a hallmark inefficiency of traditional von Neumann systems. Smooth activation functions play a central role in this context; their bounded, continuous nature aids in gradient propagation and training stability, while simultaneously reducing the dynamic range of intermediate signals, enabling inference systems to operate reliably at lower voltages and tolerate quantization and noise. These characteristics align naturally with the operational principles of analog circuits, where power scales quadratically with voltage, and with the signal constraints of CIM substrates, which often function under stringent voltage and resolution limits. In particular, CIM systems based on resistive RAM (RRAM) exemplify this synergy. In a pioneering demonstration, Yao et al. (2020) built a fully hardware-implemented five-layer convolutional neural network using one-transistor–one-memristor (1T1R) RRAM arrays, achieving more than two orders of magnitude improvement in energy efficiency over state-of-the-art GPUs while maintaining high classification accuracy on the Modified National Institute of Standards and Technology (MNIST) dataset. Similarly, Zhang et al. (2020) introduced an 8-bit CIM core using binary RRAM cells with shared successive approximation register analog-to-digital converters (SAR ADCs), reaching an effective number of bits (ENOB) of 7.26 and energy efficiency up to 0.61 tera operations per second per WATT (TOPS/W), illustrating the feasibility of inference under modest resolution and bit depth. More recently, Li Y. et al. (2023) extended this direction with M3D-LIME, a monolithically integrated 3D architecture combining analog RRAM-based CIM, digital memory, and control logic. Their system demonstrated efficient matrix–vector multiplication for feature extraction, coupled with ternary content-addressable memory (TCAM) for matching, enabling one-shot learning with 96% accuracy on the Omniglot dataset. Notably, the chip achieved an 18.3 × improvement in energy efficiency and a 2.73 × speedup over GPU baselines. These results reinforce the value of *in situ* analog computing and vertical memory-compute integration for subthreshold-compatible inference. While these implementations demonstrate strong energy savings, they also highlight analog constraints, including variability across memory cells and the need for robust calibration mechanisms.

Importantly, smooth activation functions further simplify hardware deployment of quantized networks. Continuously differentiable activations have been shown to outperform non-smooth counterparts like ReLU in noisy, low-precision environments typical of analog systems. This makes it feasible to compress architectures such as MobileNet-V3 using quantization-aware training and structured pruning, then deploy them directly onto analog CIM arrays with minimal loss in accuracy. The co-design of compression algorithms and analog-friendly activations thus forms a crucial step toward energy-scalable inference.

Beyond energy efficiency, subthreshold-inspired dynamics also enhance computational expressivity. In biological systems, subthreshold activity supports temporal integration of synaptic inputs, enabling neurons to encode not just the presence of a stimulus, but also its timing and context. This continuous signal modulation allows for richer, non-binary decision-making and probabilistic inference; capacities that are vital for robust perception and adaptive behavior. Algorithmically, smooth activation functions mirror this capability by enabling fine-grained, differentiable transformations that capture subtle input variations. These activations improve gradient flow during training, increase function smoothness, and expand the network’s capacity for learning complex mappings. Thus, subthreshold computation is not merely an energy-saving mechanism; it fundamentally expands the computational toolkit of neural systems.

### 5.2 Hierarchical sparsity and inhibitory balancing for localized efficiency

In biological neural systems, computation is not uniformly distributed across all inputs or regions of a cell. Instead, it is spatially distributed and context-dependent. Dendritic arbors, which are branch-like extensions of neurons, act as semi-autonomous processing units that filter, amplify, or suppress synaptic inputs before those signals reach the soma (Huang et al., 2022). This structure allows for input-dependent, subthreshold computation that reduces energy expenditure by confining processing to local regions of the neuron. Even passive dendrites, lacking active ion channels, can implement non-linear operations such as eXclusive OR (XOR), demonstrating that complex logic can emerge from spatial integration alone (Cazé et al., 2013). This energy-aware architecture enables neurons to avoid costly spikes unless inputs cross a carefully integrated threshold, making it a foundational mechanism for biological efficiency. Critically, inhibitory interneurons play a key role in controlling signal propagation and shaping the excitatory-inhibitory (E-I) balance in cortical networks. By modulating neuronal excitability and gating dendritic inputs, these inhibitory units prevent runaway activation and sharpen the spatial focus of computation. Inhibitory dynamics have been shown to support sparse, efficient coding in biological systems by suppressing redundant responses and enhancing selectivity.

This idea translates naturally into the design of ANNs, where structured pruning can emulate the selective refinement of dendritic computations. Instead of uniformly removing weights, structured pruning eliminates entire filters, blocks, or branches based on their contribution to the network’s performance. For example, Taylor-based importance estimation has demonstrated that as much as 40% of filters in ResNet-101 can be pruned with only a 0.02% top-1 accuracy loss (Molchanov et al., 2019). Such structured sparsity reduces not only the number of parameters but also the number of activations that need to be computed or stored, thus, bringing computational flows into closer alignment with localized, low-traffic pathways. The impact on energy consumption is especially significant for deployment on edge devices. When pruned branches are never activated, their intermediate computations can remain entirely within on-chip static random access memory (SRAM), avoiding the expensive off-chip dynamic RAM (DRAM) transfers that dominate the power budget of conventional architectures (Horowitz, 2014). This localization of memory access mimics the neuron’s own strategy of minimizing communication overhead through spatial filtering and inhibition. That said, structured pruning can introduce challenges in hardware mapping where irregular sparsity patterns may be difficult to exploit on certain edge accelerators without dedicated support. Compiler-level sparsity-aware scheduling is essential to translate these theoretical gains into practical energy savings.

Interestingly, the parallels with neurodevelopment extend even further. During early brain development, the nervous system undergoes a phase of exuberant synaptogenesis, creating a highly overconnected network, followed by synaptic pruning that eliminates redundant or inefficient connections. This exuberance-refinement cycle allows the system to first explore a broad functional space and then optimize for efficiency and specificity (Innocenti and Price, 2005). A similar phased approach in artificial networks, where dense training is followed by periodic structured pruning, can yield compact models with minimal accuracy loss while benefiting from initial overparameterization.

Incorporating hierarchical sparsity from the outset redefines pruning as a design principle, not a *post hoc* optimization. It enables networks to reduce computational complexity, memory bandwidth, and energy consumption, all while maintaining expressive power. When paired with smooth activations and subthreshold-compatible hardware, this approach offers a biologically grounded and practically viable path toward high-efficiency TinyAI.

### 5.3 Graded-activation micro-networks and temporal coding

In biological systems, temporal structure is integral to computation. Subthreshold changes in membrane potential influence the timing of action potentials, enabling neurons to encode information not only in firing rates but also in spike timing. Mechanisms such as spike-timing-dependent plasticity (STDP), phase coding, and delay-based synaptic integration highlight the importance of time-dependent dynamics. These subthreshold-driven modulations support fine-grained, context-sensitive responses that underlie learning, prediction, and rapid adaptation. Analogously, in artificial systems, integrating graded activation functions into modular micro-networks can emulate these biological strategies. Micro-networks (i.e., compact, semi-independent subnetworks embedded within larger architectures) can specialize in specific tasks or input contexts. When equipped with smooth, subthreshold-like activations, such as Swish or softplus, these micro-networks operate with controlled voltage swings and minimal activation entropy, making them highly compatible with analog and mixed-signal systems.

Architectural modularity enables selective activation: only relevant subnetworks respond to a given input, suppressing unnecessary computation. This principle mirrors the brain’s selective activation of dendritic subdomains and is especially useful for low-power edge AI applications. For instance, Konstantaropoulos et al. (2025) demonstrated dynamic activation within spiking SNN ensembles, achieving a 20 × reduction in computation with negligible accuracy loss on CIFAR-10. Datta et al. (2021) introduced a hybrid input encoding scheme that combined analog and spike-based signals, resulting in up to 125 × lower compute energy on CIFAR-100 compared to conventional approaches. Moreover, event-driven models inspired by these biological principles can maintain low idle power and initiate computation only upon detecting relevant signals, akin to the way sensory systems filter stimuli. This strategy is ideal for latency-sensitive applications like gesture recognition, anomaly detection in sensors, and auditory scene analysis. The integration of temporal coding principles into TinyAI models further expands the energy-performance trade space and enables real-time responsiveness in energy-constrained settings. Yet, designing micro-networks with dynamic routing presents trade-offs: the control overhead for activation gating can offset some energy benefits unless carefully optimized. Moreover, temporal coding architectures require precise timing and synchronization, which remain difficult to guarantee on analog substrates with high device variability.

### 5.4 Heterogeneous substrates and robustness to noise

Biological brains do not rely on a single type of neuron, signal, or circuit architecture to process information. They are constructed from a diverse array of neurons and signaling modalities, combining analog subthreshold processing, discrete spiking, neuromodulation, and local plasticity across different timescales. Subthreshold integration occurs continuously in dendrites, while action potentials represent discrete, all-or-none spikes that communicate over long distances. Ion channels vary in density and kinetics across compartments, and synapses operate on a wide range of transmission modes, from analog modulation to spike-triggered release. This intrinsic heterogeneity is not just a product of evolutionary happenstance; it is a design strategy that allows biological systems to balance energy efficiency with robustness to noise, and adaptability (Sterling and Laughlin, 2015). This distributed division of labor provides a compelling analog for future AI systems which currently operate with homogeneous logic blocks and uniform clocking, leading to inefficiencies in both computation and communication. Translating this biological heterogeneity to AI systems suggests a design approach in which different components operate on different substrates tailored to their function. For instance, compute-heavy and static layers (e.g., early convolutional filters) can be implemented using analog CIM arrays, while highly plastic or decision-critical layers (e.g., classifiers or attention heads) may be better suited to digital or spiking substrates. This division allows each computational unit to optimize for precision, energy, or speed depending on its role. A key challenge in hybrid systems lies at the interface: how to encode and decode information when passing from a subthreshold analog component to a suprathreshold digital one. In biological systems, this translation is handled by spike generation mechanisms that convert continuous membrane potentials into discrete action potentials; in engineered systems, an equivalent interface must preserve critical information while balancing energy and fidelity. Several strategies exist for this purpose. One approach is level-crossing encoding, where changes in the analog signal beyond a certain delta trigger a digital event, mimicking event-driven spikes. Another method uses time-based encoding (e.g., pulse-width modulation or time-to-first-spike schemes), where signal amplitude is converted into timing characteristics. Conversely, decoding analog signals from digital inputs often involves digital-to-analog converters (DACs) or lookup-based interpolation to restore continuous representations. Importantly, such interfaces must be co-designed with activation functions and signal ranges to ensure compatibility and minimal information loss. This encoding-decoding boundary is where biological fidelity meets engineering constraint, and optimizing it is central to realizing functional hybrid systems. These encoding strategies, however, must operate within the constraints of analog substrates, which present unique challenges, including thermal noise, device variability, and signal drift. Biological systems manage these issues through population coding, synaptic redundancy, error-corrective dynamics, and homeostatic plasticity. These principles can be adapted in AI systems via probabilistic encoding schemes, redundancy-aware training, and architectural noise tolerance. Emerging neuromorphic platforms illustrate these concepts in action. For example, Intel’s Loihi processor uses subthreshold digital logic and sparse, event-driven communication, achieving 23.6 pJ per synaptic event and 1.7 pJ per spike within a tile (Davies et al., 2018). Materials innovation is also enabling energy-efficient subthreshold firing. For instance, dual-gated MoS_2_ transistors offer synaptic-like behavior at ultra-low energy consumption of approximately 12.7 femtojoules per spike, demonstrating long-term potentiation, paired-pulse facilitation, and spike-timing-dependent plasticity (Du et al., 2020). These devices demonstrate capabilities such as excitatory postsynaptic current, paired-pulse facilitation, and spike-timing-dependent plasticity, highlighting their potential in neuromorphic computing applications (Du et al., 2020). Meanwhile, photonic accelerators have also demonstrated ultra-fast inference by performing convolutional inference at over 2 tera-operations per second with energy consumption as low as 2.5 × 10^–19^ joules per operation (Wang et al., 2022).

Yet it is not just the substrate itself that matters; it is how computation is mapped onto it. Layers with static, low-entropy weights (e.g., embeddings or early convolutions) can be evaluated in analog or photonic domains with minimal overhead. In contrast, highly plastic components such as classifiers or online learners should reside on digital or spiking substrates, where precision and rewritability are essential. To systematically exploit substrate heterogeneity, we propose a “volatility score” metric that quantifies the update frequency and sensitivity of each model component during training. Layers with high volatility would be mapped to adaptive or rewritable substrates, while stable components could be optimized on low-power analog hardware. This co-design approach mirrors the brain’s functional allocation of stable routines to hardwired pathways and fast-changing tasks to plastic circuits.

### 5.5 Energy-aware compilation, simulation, and co-design toolchains

Achieving the potential of subthreshold-compatible TinyAI requires not just new architectures and hardware, but also a supporting ecosystem of software tools that are optimized for energy efficiency. Traditional compiler optimizations often prioritize execution speed or throughput, overlooking energy implications. However, recent work has demonstrated that energy-aware compilation can produce significant efficiency gains. For instance, energy-aware register allocation using evolutionary heuristics has reduced dynamic energy consumption by up to 17.6% in real-world applications on very long instruction word (VLIW) architectures (Stuckmann et al., 2024). Selective enabling of compiler passes, rather than relying on default optimization levels, has achieved execution time reductions of 2.4% and 5.3% on ARM Cortex-M0 and M3 processors, respectively, by avoiding unnecessary transformations that increase energy use (Georgiou et al., 2018). At the runtime level, dynamic voltage and frequency scaling (DVFS) combined with task scheduling, as in the joint exploration of CPU-memory DVFS and task scheduling (JOSS) framework, has yielded up to 21.2% energy savings by co-optimizing both processor and memory energy budgets (Chen et al., 2023). These advances point to a broader opportunity: aligning the software toolchain with the physics of subthreshold and analog computation. This requires simulators and modeling tools capable of capturing non-ideal analog behaviors, mixed-precision logic, and hierarchical architectures. Frameworks like NeuroSim and AnalogNet represent early efforts in this direction, enabling co-simulation of neural network workloads and device-level analog properties. Expanding these frameworks into comprehensive design environments will be crucial for guiding model training, placement, and deployment. Yet, simulation and compiler tooling remain underdeveloped for analog and mixed-signal systems. Modeling noise, non-idealities, and mixed-precision behavior with sufficient fidelity remains a key limitation in accurately guiding design-space exploration.

On the other hand, evaluating and guiding energy-aware optimizations necessitates the adoption of metrics that accurately reflect trade-offs between energy consumption and performance. One widely used measure is the energy-delay product (EDP), defined as the product of energy consumed and execution time (EDP = Energy × Time). While EDP offers a simple composite view, it treats energy and delay as equally significant and may fail to highlight situations where a small gain in one leads to a disproportionate cost in the other. For example, two optimization strategies might yield the same EDP, yet one may halve energy at the cost of increased latency, while the other improves speed but with minimal energy savings—making EDP ill-suited as a standalone guide in many real-world settings (Roberts et al., 2017). To address these limitations, alternative metrics such as the energy-delay sum (EDS) and energy-delay distance (EDD) have been proposed. EDS expresses energy and delay as a weighted sum, allowing developers to tune the relative importance of each term according to specific application constraints, whether optimizing for edge inference, latency-critical control, or battery longevity. EDD, by contrast, measures the Euclidean distance from the origin in an energy-delay space, offering a geometric interpretation of how far a system is from the ideal (zero energy and zero delay). This framing enables a more intuitive comparison across models and configurations, encouraging selections that consistently move toward the optimal balance (Roberts et al., 2017). Integrating these metrics into cost functions for neural architecture search, training objectives, and compiler decision trees allows for principled tradeoffs between accuracy, energy, and response time. In doing so, AI systems can evolve to exhibit energy-proportional behavior more closely aligned with biological computation, completing the transition from theoretical inspiration to practical design. However, integrating these metrics into mainstream toolchains still requires broader community consensus and tool support, especially for balancing heterogeneous objectives across latency, accuracy, and energy domains.

### 5.6 Beyond efficiency: functional advantages of subthreshold-inspired designs

While energy savings are the central promise of subthreshold-inspired AI, this design philosophy also offers compelling functional benefits that extend its appeal beyond sustainability alone. For instance, analog subthreshold dynamics enable graceful handling of signal variability, leading to improved robustness under noisy or uncertain conditions. Recent work has shown that noise-aware training on analog accelerators can yield over 2 × higher resilience to non-stationary perturbations compared to conventional models, even in edge environments (Wang et al., 2025). Subthreshold integration also naturally supports temporal encoding and continuous-time computation, providing a hardware-efficient alternative to traditional recurrent architectures. Analog memristive systems and physical neural networks have demonstrated high temporal acuity in tasks such as arrhythmia detection and gesture recognition, achieving accuracies above 96% with minimal energy overhead (Stenning et al., 2022). Furthermore, subthreshold-inspired architectures offer a promising path toward few-shot learning and rapid adaptation. Neuromorphic analog networks, such as nanomagnetic reservoir systems, have recently been shown to generalize from extremely limited training data across diverse task domains, suggesting that subthreshold dynamics support meta-learning capabilities at the hardware level (Stenning et al., 2024). At the algorithmic level, smooth activations and sparse, context-sensitive pathways also enhance generalization by enforcing low-capacity representations that better resist overfitting in small-data regimes. This mirrors biological strategies that prioritize robust, low-energy computation under uncertainty. Altogether, these findings reinforce that subthreshold-inspired AI is not merely an energy-minimization strategy; it is a multidimensional design approach that enhances robustness, temporal sensitivity, few-shot generalization, and efficiency in integrated form, particularly suited for TinyAI applications in real-world, dynamic environments.

## 6 Challenges, future directions, and conclusion

While this paper outlines a compelling vision for subthreshold-inspired TinyAI, several key challenges must be addressed before these ideas can reach mainstream adoption. One of the foremost obstacles is the gap between biological computational strategies and their artificial counterparts. Subthreshold processing, although fundamental to the energy efficiency of biological systems, has not yet been effectively translated into scalable AI architectures. Current AI models remain anchored in digital logic, dominated by abrupt, high-power operations that run counter to the graded, low-voltage signaling observed in nature. Integrating subthreshold principles into artificial systems requires a fundamental redesign of activation functions, computational hierarchies, and hardware architectures. Hardware readiness is another limiting factor. While neuromorphic systems and hybrid analog-digital platforms have shown promise, they are not yet widely accessible or mature enough for deployment at scale. The analog components that are essential for subthreshold-compatible systems pose engineering challenges, including susceptibility to noise, signal degradation, and the absence of standard development environments. Moreover, there is a lack of robust simulation tools, compilers, and co-design frameworks that can support the nuanced requirements of subthreshold computation, further slowing adoption.

Nonetheless, these challenges open the door for innovative research and development. This perspective proposes that subthreshold dynamics should not be treated as a peripheral curiosity, but rather as a central design principle that can radically redefine how we build energy-efficient and context-sensitive AI systems. Our aim is to shift the conversation from isolated optimizations to a broader rethinking of AI architecture; one that aligns with the inherent efficiency of biological computation. Future research directions include the development of smooth, differentiable activation functions that mimic the continuous membrane fluctuations seen in neurons, allowing models to operate effectively at low voltages. New network architectures must incorporate spatially localized processing and hierarchical sparsity inspired by dendritic computation, reducing energy use by focusing computation only where it is most relevant. Modular, temporally sensitive micro-networks should be explored to allow for selective activation, enabling systems to respond intelligently while minimizing idle power consumption. In parallel, the creation of analog and mixed-signal CIM hardware, based on emerging memory technologies, offers a path to implement these ideas physically with drastically lower energy budgets. Although we frame this work around TinyAI, the underlying design principles are also relevant for improving the energy proportionality of large-scale AI systems, even if they do not render them “tiny.” Beyond the model and hardware levels, progress also depends on the development of energy-aware simulation platforms, training strategies, and compiler toolchains that can accurately model the behavior of subthreshold and mixed-signal components. The introduction of new metrics, such as energy-delay sum and energy-delay distance, will be crucial for evaluating trade-offs in real-world deployment scenarios, where latency and energy are often more critical than raw accuracy.

Importantly, this paper underscores the need for coordinated, interdisciplinary collaboration. This vision is reflected in Figure 2, which outlines how collaborative foundations across disciplines yield system-level efficiency and social impact through Sustainable TinyAI. Realizing the potential of subthreshold-inspired TinyAI will require tight integration between neuroscience, computer science, information systems, computer and electrical engineering, materials science, and equally, the engagement of ethicists and policymakers. As AI continues to proliferate into mobile, embedded, and edge systems, the demand for energy-efficient, explainable, and equitable solutions becomes increasingly urgent. This perspective contributes by offering a comprehensive, biologically informed framework that connects the theoretical benefits of subthreshold processing to actionable strategies in AI design. It advocates a departure from the *status quo* and calls for a new generation of systems that are not only computationally capable but also sustainable, deployable, and aligned with the energy constraints of our world.

## Data Availability

The original contributions presented in this study are included in this article/supplementary material, further inquiries can be directed to the corresponding author.
